# Neurofilament-light and contactin-1 association with long-term brain atrophy in natalizumab-treated relapsing-remitting multiple sclerosis

**DOI:** 10.1177/13524585221118676

**Published:** 2022-09-03

**Authors:** Zoë YGJ van Lierop, Samantha Noteboom, Martijn D Steenwijk, Maureen van Dam, Alyssa A Toorop, Zoé LE van Kempen, Bastiaan Moraal, Frederik Barkhof, Bernard MJ Uitdehaag, Menno M Schoonheim, Charlotte E Teunissen, Joep Killestein

**Affiliations:** Department of Neurology, Amsterdam UMC, Vrije Universiteit Amsterdam, MS Center Amsterdam, Amsterdam Neuroscience, Amsterdam, the Netherlands; Department of Anatomy and Neurosciences, Amsterdam UMC, Vrije Universiteit Amsterdam, MS Center Amsterdam, Amsterdam Neuroscience, Amsterdam, the Netherlands; Department of Anatomy and Neurosciences, Amsterdam UMC, Vrije Universiteit Amsterdam, MS Center Amsterdam, Amsterdam Neuroscience, Amsterdam, the Netherlands; Department of Anatomy and Neurosciences, Amsterdam UMC, Vrije Universiteit Amsterdam, MS Center Amsterdam, Amsterdam Neuroscience, Amsterdam, the Netherlands; Department of Neurology, Amsterdam UMC, Vrije Universiteit Amsterdam, MS Center Amsterdam, Amsterdam Neuroscience, Amsterdam, the Netherlands; Department of Neurology, Amsterdam UMC, Vrije Universiteit Amsterdam, MS Center Amsterdam, Amsterdam Neuroscience, Amsterdam, the Netherlands; Department of Radiology and Nuclear Medicine, Amsterdam UMC, Vrije Universiteit Amsterdam, MS Center Amsterdam, Amsterdam Neuroscience, Amsterdam, the Netherlands; Department of Radiology and Nuclear Medicine, Amsterdam UMC, Vrije Universiteit Amsterdam, MS Center Amsterdam, Amsterdam Neuroscience, Amsterdam, the Netherlands/Queen Square Institute of Neurology and Centre for Medical Image Computing, University College London, London, UK; Department of Neurology, Amsterdam UMC, Vrije Universiteit Amsterdam, MS Center Amsterdam, Amsterdam Neuroscience, Amsterdam, the Netherlands; Department of Anatomy and Neurosciences, Amsterdam UMC, Vrije Universiteit Amsterdam, MS Center Amsterdam, Amsterdam Neuroscience, Amsterdam, the Netherlands; Neurochemistry Laboratory, Department of Clinical Chemistry, Amsterdam UMC, Vrije Universiteit Amsterdam, Amsterdam Neuroscience, Amsterdam, the Netherlands; Department of Neurology, Amsterdam UMC, Vrije Universiteit Amsterdam, MS Center Amsterdam, Amsterdam Neuroscience, Amsterdam, the Netherlands

**Keywords:** Multiple sclerosis, natalizumab, neurofilament-light, contactin-1, MRI volumetrics

## Abstract

**Background:**

Despite highly effective treatment strategies for patients with relapsing-remitting multiple sclerosis (RRMS), long-term neurodegeneration and disease progression are often considerable. Accurate blood-based biomarkers that predict long-term neurodegeneration are lacking.

**Objective:**

To assess the predictive value of serum neurofilament-light (sNfL) and serum contactin-1 (sCNTN1) for long-term magnetic resonance imaging (MRI)–derived neurodegeneration in natalizumab-treated patients with RRMS.

**Methods:**

sNfL and sCNTN1 were measured in an observational cohort of natalizumab-treated patients with RRMS at baseline (first dose) and at 3 months, Year 1, Year 2, and last follow-up (median = 5.2 years) of treatment. Disability progression was quantified using “EDSS-plus” criteria. Neurodegeneration was measured by calculating annualized percentage brain, ventricular, and thalamic volume change (PBVC, VVC, and TVC, respectively). Linear regression analysis was performed to identify longitudinal predictors of neurodegeneration.

**Results:**

In total, 88 patients (age = 37 ± 9 years, 75% female) were included, of whom 48% progressed. Year 1 sNfL level (not baseline or 3 months) was associated with PBVC (standardized (std.) β = −0.26, *p* = 0.013), VVC (standardized β = 0.36, *p* < 0.001), and TVC (standardized β = −0.24, *p* = 0.02). For sCNTN1, only 3-month level was associated with VVC (standardized β = −0.31, *p* = 0.002).

**Conclusion:**

Year 1 (but not baseline) sNfL level was predictive for long-term brain atrophy in patients treated with natalizumab. sCNTN1 level did not show a clear predictive value.

## Introduction

Highly effective therapies for relapsing-remitting multiple sclerosis (RRMS) such as natalizumab abolish relapses and magnetic resonance imaging (MRI) activity in most patients, particularly after the first year of treatment.^1,2^ Despite highly effective therapy, ongoing (so-called “silent”) disease progression is observed in a substantial portion of patients.^3^ In explaining the underlying mechanisms, studies that focus on long-term brain atrophy as a marker of MRI-derived neurodegeneration in larger natalizumab-treated cohorts are currently lacking.^4,5^ Blood-based biomarkers that accurately reflect neurodegenerative processes are highly relevant for improved prediction of treatment response in light of both disease progression and brain atrophy.^6^

Serum neurofilament-light (sNfL) has become a well-established biomarker for neuro-axonal damage in MS, and the previous studies have shown that it has predictive value for disability, as well as brain and spinal atrophy in both relapsing and progressive MS.^7,8^ However in our previous work, sNfL failed to capture disability progression in natalizumab-treated patients with RRMS.^9^ Similar findings have been recently reported from the ASCEND cohort of secondary progressive MS patients treated with natalizumab.^10^ While sNfL is known to be a powerful tool to detect axonal loss related to acute inflammation, its prognostic value for MRI-derived neurodegeneration during highly effective therapy remains unclear.^11^

Contactin-1 (CNTN1) is a cellular adhesion molecule involved in axo–glial interaction, is thought to be released into the cerebrospinal fluid (CSF) and blood after axonal injury, and could therefore be an alternative marker for disease progression in MS. In the previous work, we have found a significant association with long-term disability progression in the same cohort^12^ and positive correlations of CSF CNTN1 level with normalized brain volume in secondary progressive multiple sclerosis (SPMS).^13^ With regard to the role of MRI scanning in explaining disease progression, earlier work has mostly focused on whole-brain atrophy.^5^ More recent work has shown the power of regional atrophy of especially deep gray matter (DGM) structures like the thalamus, which has been proposed as an important driving factor of disease progression across MS phenotypes.^14^ So far, only one relatively small cohort reported ongoing DGM atrophy in relation to disease progression in natalizumab-treated RRMS.^15^

In this study, we aim to bridge this knowledge gap and investigate the predictive value of sNfL and serum contactin-1 (sCNTN1) levels for long-term brain and thalamus atrophy, and ventricular growth in an observational cohort of closely monitored natalizumab-treated patients with RRMS.

## Methods

### Participants

Patients were selected from an ongoing prospective observational natalizumab-treated RRMS cohort, initiated in 2006 at Amsterdam UMC, location VU Medical Center. The selection was performed in November 2020, after which the database was closed for this study. As previously described, inclusion criteria were an age of 18 years or older at the time of natalizumab initiation and a minimum follow-up duration of 3 years.^12^ Natalizumab initiation was considered the baseline time point, and the last visit before natalizumab discontinuation or database closure for this project in November 2020 was considered the last follow-up time point. Clinical assessments were performed at baseline and continued on a yearly basis, and included relapse history and “EDSS-plus” assessments: a combination of the Expanded Disability Status Scale (EDSS), Timed 25-Foot Walk Test (T25FW), and 9-Hole Peg Test (9HPT).^12^ “EDSS-plus status” was determined for each subject between Year 1 and last follow-up visit, correcting for disability changes due to residual inflammation or anti-inflammatory effects of natalizumab (disability improvement) in the first year of treatment.^2,16^ Furthermore, EDSS-plus assessments within 1 year of a relapse were excluded. The EDSS-plus status was defined as “progressor” for subjects with significant worsening of either EDSS, 9HPT, or T25FW, which had to be confirmed by at least one subsequent visit. Thresholds were a 1.5, 1, or 0.5 point increase in case of a reference EDSS of 0, 1–5, or ⩾5.5, respectively, and 20% change in 9HPT or T25FW.^17^ Subjects who did not comply with these criteria were defined as “non-progressor.”

### sNfL and serum CNTN1 measurement

Blood samples were collected at baseline before the first natalizumab infusion and every 3 months onwards and processed at the Amsterdam UMC MS biobank. Centrifugation (1800*g*, 10 minutes at room temperature) was performed within 2 hours and serum samples were stored at −80°C. For this study, the following five time points were selected: baseline (prior to the first natalizumab dose), 3 months after the first dose (as less inflammation-driven re-baseline), Year 1 and Year 2 of treatment, and last follow-up under natalizumab treatment. We used the same methodologies as employed in the previous studies.^9,12^ In brief, sNfL was measured by the Simoa NF-light^®^ Advantage Kit (Quanterix, Billerica, MA, USA), and sCNTN1 was measured on a Luminex platform (Human contactin-1 Magnetic Luminex Assay, R&D systems, Minneapolis, MN, USA).

### MRI

MRI scans (including T1-, and PD/T2-weighted images) were collected on a yearly basis after the initial baseline scan (within 3 months of natalizumab initiation) or every 3 months in John-Cunningham (JC) virus seropositive patients, following the Magnetic Resonance Imaging in Multiple Sclerosis (MAGNIMS) expert panel guidelines.^18^ Radiological disease activity was defined as new/enlarged T2 hyperintense lesions and/or T1 gadolinium-enhanced (T1GE) lesions by neuroradiologists blinded to the clinical data.

### MRI image processing

As MRI data were obtained in clinical practice, subjects were scanned on multiple MRI scanners and with multiple imaging protocols. Screening of the data revealed that PD/T2-weighted images were most consistently available (in 98% of the data sets) (see Supplemental eTable 1 and eFigure 1 for details on availability).

**Figure 1.**
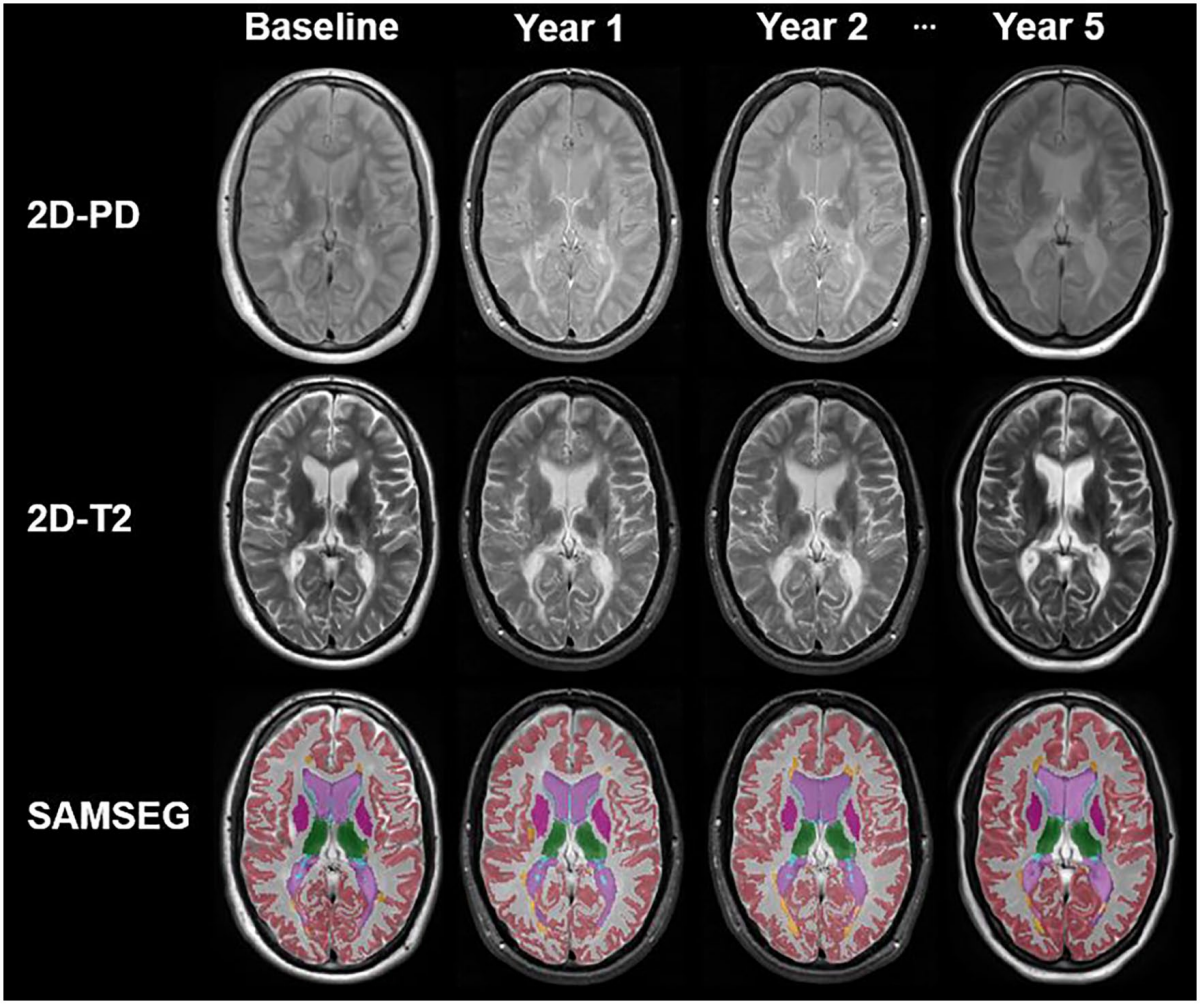
Example of longitudinal brain segmentation on 2D PD/T2-weighted images with sequence-adaptive segmentation method (SAMSEG). red: cortex; white: white matter; purple: ventricle; green: thalamus; dark purple: putamen; orange: lesions.

Because of the variation in imaging protocols, brain tissue segmentation was performed with the MS-specific longitudinal version of the Sequence Adaptive Multimodal Segmentation (SAMSEG) method, recently provided in the open-source neuroimaging package FreeSurfer 7.1.1 (Figure 1).^19,20^ The longitudinal pipeline of SAMSEG is specifically designed for handling data from different origins by adapting to the different MRI protocols and making use of the shared information across repeated scans of the same subject.^20^ In addition, the MS version of the pipeline automatically segments white matter lesion along with other brain structures, such that lesion filling is not required.

SAMSEG requires all input images to be co-registered to the same image space. Therefore, an average PD/T2-weighted template was created across all time points for each subject with FSL midtrans (part of the functional MRI of the brain (FMRIB) Software Library (FSL; version 5.0.4, http://fsl.fmrib.ox.ac.uk)) and all PD/T2-weighted images were rigidly registered to this average subject-specific template. To ensure a consistent voxel size and orientation across subjects, the average template was constructed in standard 1 mm Montreal Neurological Institute brain template (MNI) space.

After running SAMSEG on the standardized images, total brain volume, lateral ventricle volume, thalamic volume, and white matter lesion volume were derived. Volumes were expressed as fractions of mean intracranial volume (ICV) across all time points, resulting in brain parenchymal fraction (BPF), lateral ventricle fraction (VF), thalamus fraction (TF), and lesion fraction (LF), respectively.

Annualized percentage brain volume change (PBVC) was determined for each subject by performing linear regression on the measurement results of all time points between Year 1 and the last visit. Measurements in the year after treatment initiation were not taken into account to rule out the potential effects of pseudo-atrophy.^21,22^ The same procedure was followed to calculate annualized ventricle volume change (VVC) and thalamus volume change (TVC).

### Statistical analyses

Statistical analyses were performed with IBM SPSS Statistics Version 26.0 (IBM Corp., Armonk, NY, USA) and R statistical software version 4.0.3. As previously described, clinical and radiological characteristics were compared between EDSS-plus progressors and non-progressors using chi-square test for categorical variables (gender, occurrence of relapse(s), or radiological disease activity) and Mann–Whitney *U* test to compare the non-normally distributed continuous variables (age, disease duration, number of relapses, T1GE lesion numbers, EDSS, 9HPT, and T25FW).^12^

A linear mixed-effects model was used to investigate the longitudinal associations between MRI volumes (BPF, VF, TF, and LF, respectively) and blood biomarker levels (sNfL and sCNTN1). Time points included in the linear mixed-effects model for both biomarker levels and MRI volumes were Year 1, Year 2, and last follow-up. For all linear mixed-effects models, time, interaction of time with MRI volume, and disease duration were included as fixed effects, and subject as random-effect to adjust for the within-subject effect of repeated measures. To account for non-normal distribution, blood biomarker levels were log-transformed and disease duration was square root transformed.

For the design of prediction models for future PBVC, VVC, and TVC, the first step was to identify the best candidate predictors by carrying out univariate regression analyses including three categories (1) clinical and radiological disease activity variables during the first year of treatment (to identify possible inflammation-driven predictors of neurodegeneration), (2) Year 1 disability and MRI volume measures and (3) the cross-sectional biomarker levels at baseline, 3 months, and Year 1.

Candidate predictors that showed significant associations in the univariate analyses were subsequently included in a step-wise multivariate regression analysis. Forward selection (*p*-value < 0.05) was used to determine the best possible prediction model for PBVC, VVC, and TVC. All statistical analyses were corrected for sex and age at baseline. A *p*-value of <0.05 was considered statistically significant for all analyses.

### Ethical considerations

The Institutional Review Board (Medical and Biobank Ethics Committee of Amsterdam UMC, location VUmc) approved the use of routine medical files for research purposes (registration no. 2016.554). All subjects gave written informed consent for the collection and use of medical data and biological fluids for research purposes. This study adhered to the ethical principles of the Declaration of Helsinki.

### Data availability

Anonymized data not published within this article will be made available upon reasonable request from a qualified investigator.

## Results

### Baseline and follow-up characteristics

**Figure 2.**
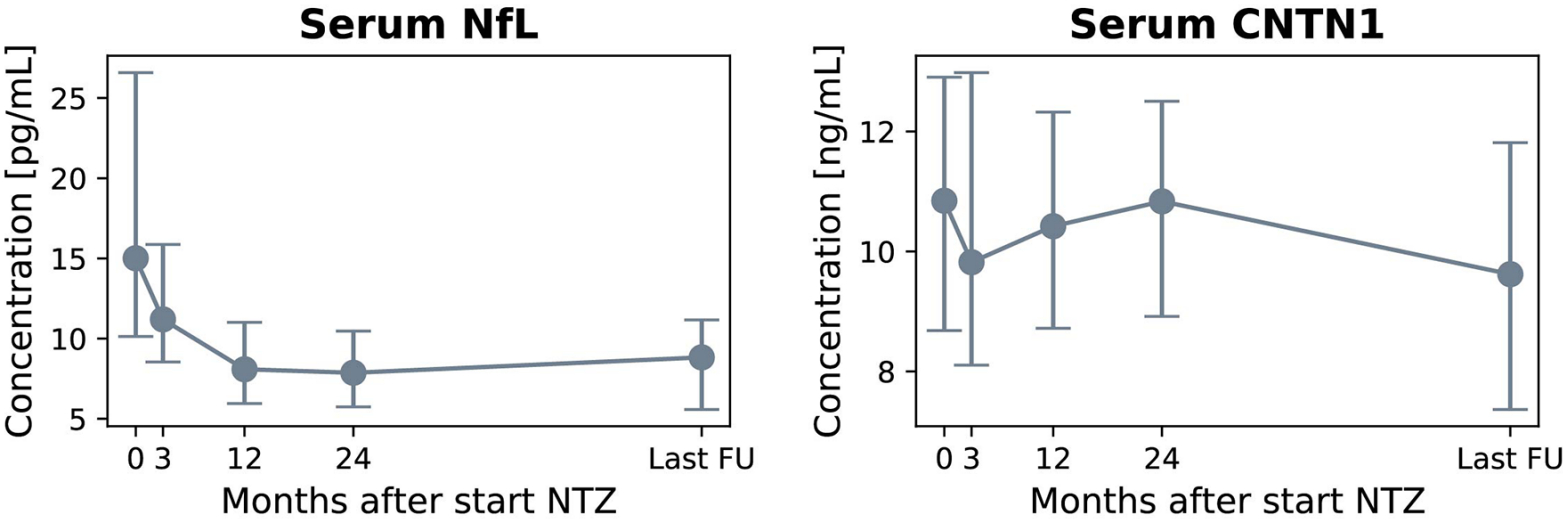
Median serum NfL and serum CNTN1 levels for the total cohort (*n* = 88) at each time point. Natalizumab initiation is regarded baseline time point, followed by 3, 12 (Year 1) and 24 months (Year 2) of treatment, and last follow-up (median = 5.2 years (4.3–6.8)). Interquartile ranges are presented by the vertical bars. NfL: neurofilament-light; CNTN1: contactin-1.

Based on our inclusion criteria, a total of 89 natalizumab-treated RRMS patients were selected as previously described.^12^ One patient was excluded because of MRI artifacts complicating volume measurement, resulting in a total number of 88 patients included in the current follow-up study (age 36 ± 8.6 years, 75% female) with a median follow-up duration of 5.2 years (interquartile range (IQR) = 4.3–6.8). Other baseline and follow-up clinical and radiological characteristics, and sNfL and sCNTN1 levels are summarized in Table 1 and Figure 2. With regard to disease activity measures, at baseline, median relapse rate 1-year pre-baseline was 1 (IQR = 1–2), and 65% of patients had MRI activity at baseline (median number of T1GE lesions of 2 (IQR = 0–6)). During Year 1 of follow-up, 15% of patients experienced a relapse, and 30.2% showed evidence of radiological activity on the Year 1 brain MRI scan. Between Year 1 and last follow-up, 9.1% of patients experienced a relapse and 8.0% of patients showed radiological disease activity.

**Table 1.** Baseline and follow-up clinical and radiological characteristics, sNfL, and sCNTN1 levels.

Baseline and follow-up characteristics	Total (*n* = 88)
Females (%)	75
Age at baseline (years)	36.8 ± 8.6
Disease duration at baseline (years)	7.4 (3.8–12.1)
Time between baseline and last follow-up (years)	5.2 (4.3–6.8)
Clinical disability measures, Year 1^a^	
EDSS	3.5 (2.5–5.0)
9HPT (seconds)	21.6 (19.8–26.2)
T25FW (seconds)	4.9 (3.9–7.2)
EDSS-plus progression, Year 1—last follow-up	
Yes (%)	48
No (%)	52
Relapses	
1-Year pre-baseline (number)	1 (1–2)
Baseline—Year 1 (%)	15
Year 1—FU (%)	9.1
MRI activity^b^	
Baseline (%)/T1GE lesions (number)	65/2 (0–6)
Baseline—year 1 (%)	30.2
Year 1—FU (%)	8.0
sNfL (pg/mL)	
Baseline	15.0 (10.12–27.70)
3 months	11.2 (8.43–16.13)
12 months	8.1 (5.95–11.02)
24 months	7.9 (5.74–10.54)
Last follow-up	8.8 (5.58–11.26)
sCNTN1 (ng/mL)	
Baseline	10.9 (8.68–12.92)^c^
3 months	9.8 (8.09–13.07)
12 months	10.4 (8.66–12.37)
24 months	10.8 (8.83–12.55)
Last follow-up	9.6 (7.29–11.86)
Volumes at baseline (mL)	
Lesions	8 (3.3–16)
Whole brain	1011 ± 112
Ventricles	40.5 ± 12.4
Thalamus	10.7 ± 1.6
Fraction of intracranial volume Year 1 (%)^d^	
BPF	72 ± 3.3
VF	2.9 ± 0.9
TF	0.8 ± 0.1
LF	0.8 ± 0.09
Annualized change (%), baseline—Year 1	
Whole brain	−0.58 ± 2.3
Ventricles	1.9 ± 5.7
Annualized change (%), Year 1—FU	
Whole brain	−0.22 ± 1.1
Ventricles	0.25 ± 1.9
Thalamus	−0.27 ± 1.3

EDSS: Expanded Disability Status Scale; 9HPT: 9-Hole Peg Test; T25FW: Timed 25-Foot Walk Test; FU: follow-up; MRI: magnetic resonance imaging; sNfL: serum neurofilament-light; sCNTN1: serum contactin-1; T1GE: T1 gadolinium-enhancement; BPF: brain parenchymal fraction; VF: ventricle fraction; TF: thalamus fraction; LF: lesion fraction.

Mean values are presented with ±standard deviation and median values with (interquartile range).

a Year 1 clinical disability measures are displayed instead of baseline, to take into account the disability improvement in the first year after natalizumab initiation due to anti-inflammatory effects.

b MRI activity was defined as new/enlarged T2 lesions and/or T1 gadolinium-enhancing (T1GE) lesions.

c Comparing all characteristics between EDSS-plus progressors (*n* = 42) and non-progressors (*n* = 46), only baseline sCNTN1 level was significantly lower in progressors compared to non-progressors (*p* = 0.025).

d Normalized measurements of FreeSurfer are given as unit-less tissue fractions of intracranial volume (ICV) in percentages (%).

Progression in EDSS scores was found in 30% of patients. According to EDSS-plus criteria (i.e. also including T25FW and 9HPT), disability progression was established in 42 patients (i.e. progressors, 48%).

### Disability progressors versus non-progressors

Clinical and radiological characteristics (Table 1) were compared between EDSS-plus progressors and non-progressors. Relapse rates and MRI activity at baseline, in the first year of treatment, and between Year 1 and last follow-up visits showed no significant differences between these groups. With regard to the biomarkers, only baseline CNTN1 level was significantly lower in EDSS-plus progressors (9.71 ng/mL, IQR = 8.12–12.08, *p* = 0.025) compared to non-progressors (11.58 ng/mL, IQR = 9.87–13.64), as previously described.^9,12^ With regard to the MRI volumes at baseline and annualized volume changes, no significant differences were found between progressors and non-progressors.

### Longitudinal biomarker levels versus MRI volume changes

Taking into account differences between subjects and follow-up duration, linear mixed-effects models showed that, next to longer disease duration at baseline (*p* = 0.005), an increase in sNfL level over time (*p* = 0.005) between Year 1 and last follow-up was associated with a decrease in whole-brain volume in the same period (Table 2 and Supplemental eFigure 3). An increase in sNfL level was also significantly associated with a decrease in thalamus volume (*p* = 0.031). Ventricular and lesion volume did not show any longitudinal associations with sNfL or sCNTN1 levels.

**Table 2.** Linear mixed-effects models and parameter estimates for brain parenchymal fraction (BPF), ventricle fraction (VF), thalamus fraction (TF), and lesion fraction (LF).

Brain parenchymal fraction					
AIC = 537			AIC = 546		
Effects	Std. β	*p*-value	Effects	Std. β	*p*-value
Age	−0.09 (−0.29 to 0.11)	0.389	Age	−0.09 (−0.28 to 0.11)	0.383
Female sex	0.13 (−0.28 to 0.54)	0.527	Female sex	0.16 (−0.24 to 0.56)	0.436
Disease duration	−0.29 (−0.49 to −0.09)	**0.005**	Disease duration	−0.28 (−0.48 to −0.09)	**0.005**
Log sNfL	0.05 (−0.07 to 0.17)	0.444	Log sCNTN1	0.05 (−0.03 to 0.13)	0.242
Time	−0.19 (−0.26 to −0.12)	**<0.001**	Time	−0.22 (−0.29 to −0.15)	**<0.001**
Log sNfL × time	−0.09 (−0.16 to −0.03)	**0.005**	Log sCNTN1 × time	−0.04 (−0.12 to 0.03)	0.252
Ventricular fraction					
AIC = 217			AIC = 224		
Effects	Std. β	*p*-value	Effects	Std. β	*p*-value
Age	0.11 (−0.12 to 0.34)	0.364	Age	0.10 (−0.13 to 0.33)	0.371
Female sex	−0.13 (−0.61 to 0.34)	0.574	Female sex	−0.14 (−0.61 to 0.33)	0.560
Disease duration	0.22 (−0.01 to 0.46)	0.060	Disease duration	0.22 (−0.01 to 0.46)	0.060
Log sNfL	−0.01 (−0.06 to 0.04)	0.790	Log sCNTN1	−0.01 (−0.04 to 0.02)	0.511
Time	0.03 (0.01 to 0.05)	**0.014**	Time	0.03 (0.01 to 0.05)	**0.010**
Log sNfL × time	0.02 (−0.01 to 0.04)	0.147	Log sCNTN1 × time	−0.01 (−0.04 to 0.01)	0.428
Thalamus fraction					
AIC = 319			AIC = 332		
Effects	Std. β	*p*-value	Effects	Std. β	*p*-value
Age	0.12 (−0.1 to 0.34)	0.289	Age	0.13 (−0.09 to 0.35)	0.245
Female sex	0.22 (−0.24 to 0.68)	0.343	Female sex	0.24 (−0.21 to 0.7)	0.289
Disease duration	−0.36 (−0.59 to −0.14)	**0.002**	Disease duration	−0.35 (−0.57 to −0.13)	**0.002**
Log sNfL	0.08 (0.01 to 0.15)	**0.020**	Log sCNTN1	0.02 (−0.02 to 0.06)	0.280
Time	−0.10 (−0.13 to −0.06)	**<0.001**	Time	−0.09 (−0.12 to −0.06)	**<0.001**
Log sNfL × time	−0.01 (−0.04 to 0.02)	0.645	Log sCNTN1 × time	0.00 (−0.03 to 0.04)	0.774
Lesion fraction					
AIC = 350			AIC = 346		
Effects	Std. β	*p*-value	Effects	Std. β	*p*-value
Age	0.01 (−0.22 to 0.23)	0.956	Age	0.01 (−0.2 to 0.24)	0.891
Female sex	0.29 (−0.16 to 0.74)	0.209	Female sex	0.28 (−0.17 to 0.73)	0.219
Disease duration	0.28 (0.05 to 0.5)	**0.016**	Disease duration	0.27 (0.05 to 0.5)	**0.018**
Log sNfL	0.02 (−0.05 to 0.1)	0.588	Log sCNTN1	−0.01 (−0.05 to 0.03)	0.676
Time	−0.06 (−0.1 to −0.03)	**<0.001**	Time	−0.06 (−0.1 to −0.03)	**<0.001**
Log sNfL × time	−0.01 (−0.05 to 0.02)	0.500	Log sCNTN1 × time	0.00 (−0.04 to 0.03)	0.794

AIC: Akaike information criteria; sNfL: serum neurofilament-light; sCNTN1: serum contactin-1; Std. β: standardized β.

For both biomarker levels and MRI volumes, time points included in the models were Year 1, Year 2, and last follow-up. A *p*-value of <0.05 was considered statistically significant and indicated in bold. An increase in sNfL level over time between Year 1 and last follow-up was associated with a decrease in BPF in the same period (*p* = 0.005). Higher sNfL level was also significantly associated with a lower TF (*p* = 0.020). VF and LF did not show any longitudinal associations with sNfL or sCNTN1 levels.

### Univariate relationships between blood biomarker levels and MRI volume changes

**Figure 3.**
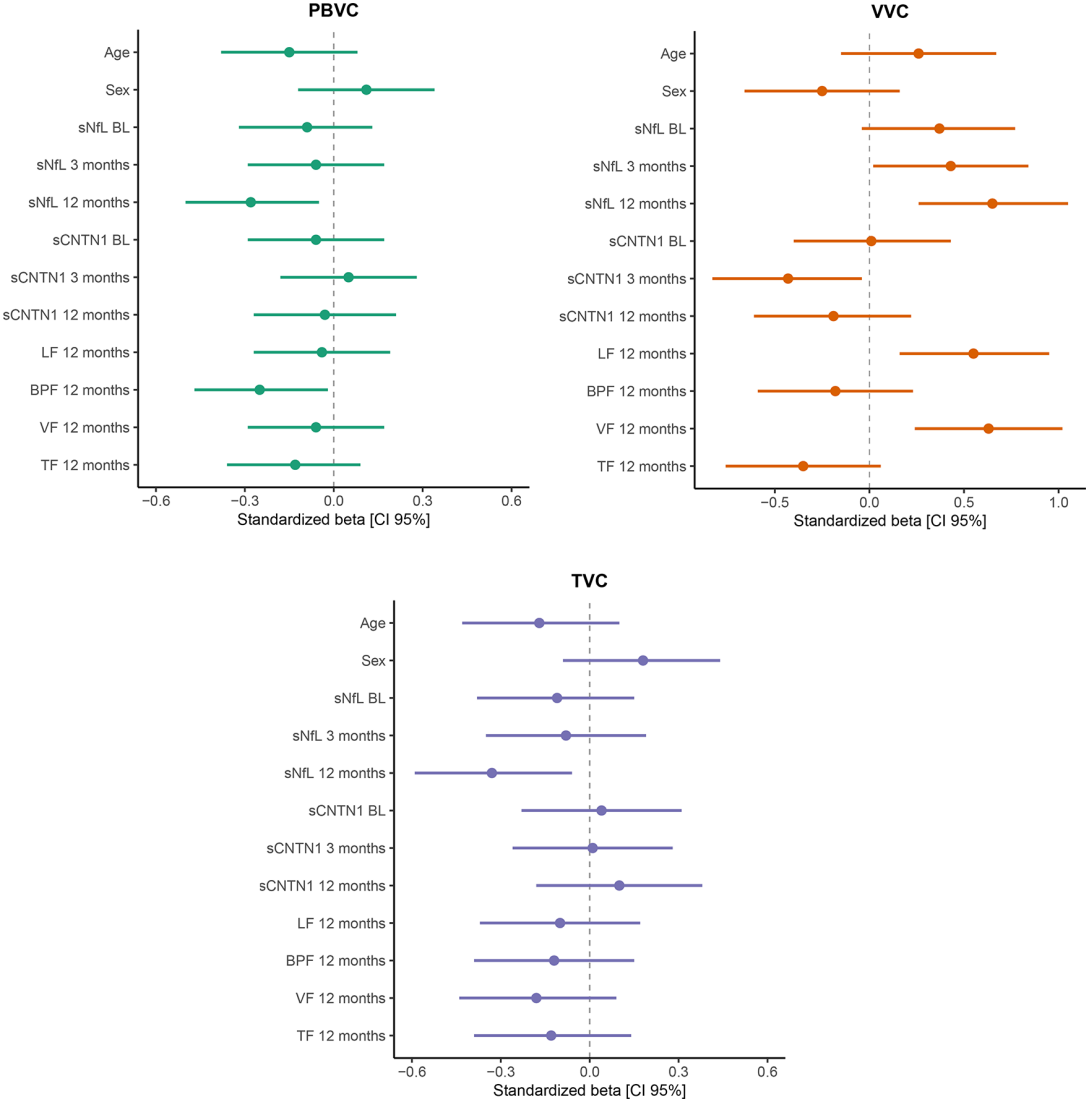
Identifying predictors of annualized percentage brain volume change (PBVC), ventricular volume change (VVC), thalamus volume change (TVC). Univariate linear regression analyses were applied, where a standardized beta with a 95% confidence interval (95% CI) that does not include zero corresponds to a statistically significant association (*p*-value < 0.05). Only the variables age, sex, biomarker levels during Year 1, and MRI volumes at Year 1 were selected to include in these plots (complete results on the univariate regression analyses are presented in Supplemental eTable 2). Volume changes were calculated between Year 1 and follow-up (natalizumab initiation is regarded baseline time point). Levels of sNfL and sCNTN1 were log-transformed. sNfL: serum neurofilament-light; sCNTN1: serum contactin-1; EDSS: Expanded Disability Status Scale; T25FW: Timed 25-Foot Walk Test; 9HPT: 9-Hole Peg Test; FU: follow-up; T1GE: T1 gadolinium-enhancement; BPF: brain parenchymal fraction; VF: ventricle fraction; TF: thalamus fraction; LF: lesion fraction.

The univariate models to identify candidate predictors of annualized PBVC, VVC, and TVC between year 1 and last follow-up MRI scan are shown in Figure 3 and Supplemental eTable 2. High year 1 sNfL level was a predictor of worse PBVC (standardized (std.) β = −0.257, *p* = 0.016), worse TVC (std. β = −0.259, *p* = 0.016), and worse VVC (std. β = 0.338, *p* = 0.001). Baseline sNfL was not associated with volume change, while sNfL level at 3 months only showed a weak association with VVC (std. β = 0.22, *p* = 0.04). In other words, Year 1 sNfL level (not baseline or 3 months levels) predicted annualized whole-brain, thalamus, and ventricular volume changes. For sCNTN1, only the 3 months level was a significant predictor of VVC (std. β = −0.230, *p* = 0.033), while baseline and Year 1 levels did not show an association for MRI volume changes. Among the remaining clinical and radiological characteristics assessed after the first year of treatment, no significant associations with MRI volume changes were found (Supplemental eTable 2).

### Step-wise linear regression of MRI volume change

The results of the step-wise linear regression analyses are shown in Table 3. The model for worse PBVC (adjusted (adj.) *R*^2^ = 0.12, *F* = 3.9, *p* = 0.006) consisted of higher Year 1 sNfL level (std. β = −0.264, *p* = 0.013) and lower BPF at Year 1 (std. β = −0.275, *p* = 0.009). Larger ventricular growth (adj. *R*^2^ = 0.28, *F* = 7.4, *p* < 0.001) was predicted by female sex (std. β = −0.198, *p* = 0.039), higher Year 1 sNfL (std. β = 0.356, *p* < 0.001), lower 3 months CNTN1 (std. β = −0.306, *p* = 0.002), and higher Year 1 LF (std. β = 0.250, *p* = 0.011). Finally, the prediction model for TVC (adj. *R*^2^ = 0.065, *F* = 3.0, *p* = 0.036) included Year 1 sNfL level (std. β = 0.250, *p* = 0.011) as the only significant predictor.

**Table 3.** Best prediction models for annualized percentage brain volume change (PBVC), ventricular volume change (VVC), and thalamus volume change (TVC).

PBVC Year 1—FU, adj. *R*^2^ = 0.12, *F* = 3.9, *p* = 0.006		
Predictors	Std. β	*p*-value
Age	−0.154	0.148
Female sex	0.125	0.221
Log sNfL at 12 months	−0.264	**0.013**
BPF Year 1	−0.275	**0.009**
VVC year 1—FU, adj. *R*^2^ = 0.28, *F* = 7.4, *p* < 0.001		
Predictors	Std. β	*p*-value
Age	0.110	0.254
Female sex	−0.198	**0.039**
Log sNfL at 12 months	0.356	**0.000**
Log sCNTN1 at 3 months	−0.306	**0.002**
Log LF Year 1	0.250	**0.011**
Thalamus year 1—FU, adj. *R*^2^ = 0.065, *F* = 3.0, *p* = 0.036		
Predictors	Std. β	*p*-value
Age	−0.088	0.415
Female sex	0.158	0.136
Log sNfL at 12 months	−0.244	**0.025**

sNfL: serum neurofilament-light; sCNTN1: serum contactin-1; BPF: brain parenchymal fraction; LF: lesion fraction; PBVC: percentage brain volume change; VVC: ventricular volume change; TVC: thalamus volume change.

Multivariate linear regression with forward selection procedure (cut-off *p*-value < 0.05) was used to establish the best prediction model for the different MRI volume changes. For the individual predictors in the models, a p-value of <0.05 was considered statistically significant and indicated in bold. Natalizumab initiation is regarded baseline time point, and volume changes were calculated between Year 1 and last follow-up (median = 5.2 years (4.3–6.8)) to correct for pseudo-atrophy.

## Discussion

This study investigated the predictive value of sNfL and sCNTN1 levels for MRI-derived neurodegeneration in an observational cohort of natalizumab-treated RRMS patients followed for a median of 5 years. The main findings of this study were that long-term brain and thalamus atrophy were best predicted by sNfL level measured after 1 year of treatment and MRI volumes, but not by baseline or 3 months sNfL levels nor sCNTN1 levels.

Stronger increases in sNfL levels between Year 1 and follow-up showed a significant intra-individual association with whole-brain and thalamus atrophy, but not with lesion volume. These findings indicate that the predictive value of sNfL for long-term atrophy under natalizumab treatment is masked in the first year of treatment, possibly by inflammatory effects, and that only later the predictive value of sNfL is driven by neurodegeneration in the context of disease progression during highly effective treatment. The observations in our study are in line with a fingolimod-treated cohort study, which reports that sNfL levels measured 1 and 2 years after treatment initiation have higher prognostic value for long-term disability and brain volume loss compared to baseline sNfL level.^23^ Furthermore, our results confirm the strong predictive value of sNfL for future neuro-axonal loss found in a cohort study of patients without disease-modifying treatment (DMT) as well as patients using first-line DMTs.^7^ Albeit study design and population differed, our findings also connect to those of a recent study of MS patients who started follow-up within 5 years of disease onset and continued for 10 years, which found that sNfL levels at 1 and 2 years of follow-up were associated with the thalamus volume at 10 years.^24^ More advanced quantifications of axonal loss in the white matter might provide additional information, for instance, using diffusion-weighted imaging. Furthermore, the explained variance of the different models is quite low, illustrating the need for additional biomarkers and the use of other (e.g. machine learning) methods to compose more powerful prediction models for MRI-derived neurodegeneration during natalizumab treatment.

With regard to sCNTN1, this candidate biomarker was not related to whole-brain or thalamic changes but only significantly predicted ventricular growth and only using the level measured after 3 months of treatment. Since ventricular growth is considered a robust indicator of both white and gray matter atrophy,^4^ this could indicate that sCNTN1 is a potential, yet weak predictor of MRI-derived neurodegeneration, and that it is less sensitive compared to sNfL. However, this result should be interpreted with caution, since the significance level of sCNTN1 at 3 months was close to the statistical threshold and we did not find any significant longitudinal associations between sCNTN1 and other MRI volumetrics. Furthermore, the previously reported association of baseline sCNTN1 to long-term disability progression in the same cohort^12^ was not confirmed by the current radiological outcomes. Another explanation could be that the association of CNTN1 to long-term disability progression is driven by neurodegeneration in a specific region outside the scope of this study, for example, the cortex, other DGM regions, or the spinal cord.^25^ Therefore, future studies measuring atrophy in more central nervous system (CNS) regions are needed to shed more light on the added value of sCNTN1 to predict radiological outcome measures.

In this natalizumab-treated cohort, no differences in brain atrophy rates over 5 years were found between EDSS-plus progressors and non-progressors. sNfL levels were associated with brain atrophy; however, sNfL failed to capture EDSS-plus progression in the studied cohort.^9^ Our findings could illustrate a lack of sensitivity of EDSS-plus assessments in the context of neurodegeneration. This is in accordance with another 3-year longitudinal study investigating natalizumab-treated patients, that also reported only a marginal association between disability changes and whole-brain atrophy.^26^ An alternative explanation could again include the role of smaller regions of DGM atrophy and the spinal cord, which have been shown to mainly drive disability progression across MS phenotypes.^14,25^

Strengths of this study include that we took into account the changes in disability and MRI volumes that occur in the first year after initiation of natalizumab treatment, which are more likely related to mechanisms of active inflammation rather than mechanisms underlying disease progression. In addition, we used well-validated and sensitive tests for biomarker analysis. Furthermore, the use of the longitudinal SAMSEG method enabled us to obtain volume changes from routine two-dimensional (2D) dual-echo T2-weighted scans acquired in standard clinical routine. To our knowledge, the longitudinal SAMSEG method has not been applied in a real-world MS cohort before. However, we acknowledge several possible limitations. First, in the first year of treatment, we did not select additional time points for biomarker analysis next to the 3 months time point. Furthermore, although the used segmentation method was designed to be robust against differences in scanners, this method has not been widely validated for longitudinal studies using clinical MRI data. The heterogeneity in acquisition protocols may have introduced noise in the calculated atrophy measures, especially in this relatively small cohort. Second, due to using real-world clinical MRI data, we were limited to the usage of 2D PD/T2-weighted scans. Three-dimensional (3D)-T1-weighted scans are still the current standard to assess atrophy, so further validation on 2D PD/T2-weighted scans is required. Third, the assessed MRI volumes were limited to global measures of total brain volume, ventricular volume, and thalamus, since image quality did not allow accurate segmentation of cortex and smaller DGM regions.

To conclude, this study demonstrated that the predictive value of sNfL for long-term atrophy in natalizumab-treated RRMS patients is masked by inflammatory effects in the first year of treatment, and only later is driven by neurodegeneration in the context of disease progression during highly effective therapy from which point it has better predictive value compared to sCNTN1 and other standard clinical and radiological characteristics.

## Supplemental Material

sj-docx-1-msj-10.1177_13524585221118676 – Supplemental material for Neurofilament-light and contactin-1 association with long-term brain atrophy in natalizumab-treated relapsing-remitting multiple sclerosis Click here for additional data file. Supplemental material, sj-docx-1-msj-10.1177_13524585221118676 for Neurofilament-light and contactin-1 association with long-term brain atrophy in natalizumab-treated relapsing-remitting multiple sclerosis by Zoë YGJ van Lierop, Samantha Noteboom, Martijn D Steenwijk, Maureen van Dam, Alyssa A Toorop, Zoé LE van Kempen, Bastiaan Moraal, Frederik Barkhof, Bernard MJ Uitdehaag, Menno M Schoonheim, Charlotte E Teunissen and Joep Killestein in Multiple Sclerosis Journal
