# Supplementary material for: Neurofilament-light and contactin-1 association with long-term brain atrophy in natalizumab-treated relapsing-remitting multiple sclerosis
Source: Mult Scler. 2022 Sep 3;28(14):2231–42. doi: 10.1177/13524585221118676 (PMC9679802; doi:10.1177/13524585221118676)
Supplement: sj-docx-1-msj-10.1177_13524585221118676 – Supplemental material for Neurofilament-light and contactin-1 association with long-term brain atrophy in natalizumab-treated relapsing-remitting multiple sclerosis [file sj-docx-1-msj-10.1177_13524585221118676.docx]

**SUPPLEMENT**

|  |  | PD | | | T2 | | |  |
| --- | --- | --- | --- | --- | --- | --- | --- | --- |
| *MRI scanner* | *N=684 scans (%)* | *TR*  *(ms)* | *TE*  *(ms)* | *FA*  *(°)* | *TR*  *(ms)* | *TE*  *(ms)* | *FA*  *(°)* | *Slice thickness*  *(mm)* |
| Siemens Sonata 1.5 T | 41.5% | 2700 | 45 | 90 | 2700 | 90 | 90 | 5 |
| GE Signa HDxt 1.5 T | 36.3% | 3924 | 26 | 90 | 3924 | 104 | 90 | 5 |
| Toshiba Titan 3.0 T | 10.3% | 2000 | 45 | 90 | 4100 | 72.5 | 90 | 5 |
| Siemens Avanto 1.5 T | 7.8% | 3000 | 25 | 90 | 3000 | 86 | 90 | 5 |
| Siemens Magnetom Vision 1.5 T | 1.5% | 2700 | 45 | 90 | 2700 | 90 | 90 | 5 |
| Siemens Magnetom Expert 1.5 T | 1.2% | 2700 | 45 | 90 | 2700 | 90 | 90 | 5 |
| GE Discovery MR750 3.0 T | 0.8% | 8662 | 23 | 111 | 8662 | 108 | 111 | 3 |
| Philips Ingenuity 1.5 T | 0.7% | 3867 | 19 | 90 | 3867 | 100 | 90 | 3 |

**eTable 1**. Overview of the MRI scanners that were used this prospective observational cohort study (a total of 684 MRI scans in 88 subjects) and the sequence parameters of the PD/T2 images.


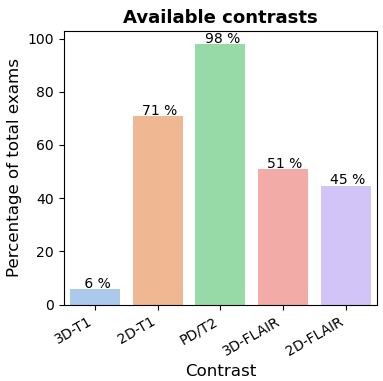


**eFigure 1**. Overview of the MRI contrasts available in this prospective observational cohort study (a total of 684 MRI scans in 88 subjects).

| Univariate analysis | PBVC year 1 - FU | | VVC year 1 – FU | | TVC year 1 - FU | |
| --- | --- | --- | --- | --- | --- | --- |
| *Predictors* | *Stand. β* | *p-value* | *Stand. β* | *p-value* | *Stand. β* | *p-value* |
| Age | -0.14 | 0.20 | 0.14 | 0.21 | -0.13 | 0.22 |
| Female sex | 0.10 | 0.34 | -0.13 | 0.22 | 0.14 | 0.19 |
| sNfL baseline | -0.09 | 0.41 | 0.19 | 0.07 | -0.09 | 0.40 |
| sNfL 3 months | -0.06 | 0.60 | **0.22** | **0.04*** | -0.07 | 0.55 |
| sNfL year 1 | **-0.26** | **0.02*** | **0.34** | **0.001*** | **-0.26** | **0.02*** |
| sCNTN1 baseline | -0.06 | 0.58 | 0.01 | 0.94 | 0.03 | 0.76 |
| sCNTN1 3 months | -0.05 | 0.67 | **-0.23*** | **0.03** | 0.01 | 0.94 |
| sCNTN1 year 1 | -0.04 | 0.81 | -0.10 | 0.36 | 0.08 | 0.48 |
| EDSS Year 1 | -0.16 | 0.14 | 0.18 | 0.09 | -0.03 | 0.79 |
| T25FW Year 1 | -0.03 | 0.78 | 0.12 | 0.29 | 0.10 | 0.39 |
| 9HPT Year 1 | 0.05 | 0.70 | 0.22 | 0.06 | 0.05 | 0.66 |
| EDSS+ progressor | -0.05 | 0.63 | 0.15 | 0.17 | -0.16 | 0.14 |
| Relapses 1 year pre-baseline | 0.06 | 0.61 | -0.03 | 0.77 | 0.03 | 0.79 |
| Relapses baseline – year 1 | 0.02 | 0.84 | 0.001 | 0.99 | 0.08 | 0.44 |
| Relapses year 1 - FU | 0.14 | 0.19 | -0.08 | 0.45 | 0.12 | 0.25 |
| MRI activity^1^ baseline | -0.17 | 0.22 | 0.16 | 0.23 | -0.18 | 0.20 |
| T1GE lesion number baseline | 0.16 | 0.13 | -0.13 | 0.23 | 0.09 | 0.43 |
| MRI activity baseline - year 1 | 0.17 | 0.11 | -0.12 | 0.29 | -0.01 | 0.79 |
| MRI activity year 1 - FU | 0.09 | 0.40 | 0.05 | 0.64 | 0.08 | 0.48 |
| LF year 1 | -0.08 | 0.47 | **0.29** | **0.01*** | -0.05 | 0.68 |
| BPF year 1 | **-0.23** | **0.03*** | -0.09 | 0.39 | -0.09 | 0.38 |
| VF year 1 | -0.06 | 0.59 | **0.33** | **0.002*** | -0.14 | 0.19 |
| TF year 1 | -0.12 | 0.25 | -0.18 | 0.09 | -0.10 | 0.35 |

**eTable 2.** **Identifying predictors of annualized percentage brain volume change (PBVC), ventricular volume change (VVC) and thalamus volume change (TVC).**Univariate linear regression analysis was used and all available clinical, radiological and biomarker levels in the first year of natalizumab treatment were included. Natalizumab initiation is regarded baseline timepoint and MRI volume changes were calculated between year 1 and last follow-up to correct for pseudo-atrophy. Levels of sNfL and sCNTN1 were log-transformed. Significant predictors are indicated in bold, defined by a p-value <0.05 (*).

^1^MRI activity is defined as new/enlarged T2 hyperintense lesions and/or T1 gadolinium-enhancing (T1GE) lesions (for baseline MRI activity, this was compared to the first available pre-baseline brain MRI scan ).

*sNfL = serum neurofilament-light; sCNTN1 = serum contactin-1; EDSS = expanded disability status scale; T25FW = timed 25-foot walk test; 9HPT = 9-hole peg test, FU = follow-up; T1GE = T1 gadolinium-enhancement; BPF = brain parenchymal fraction; VF = ventricle fraction; TF = thalamus fraction; LF = lesion fraction.*

*
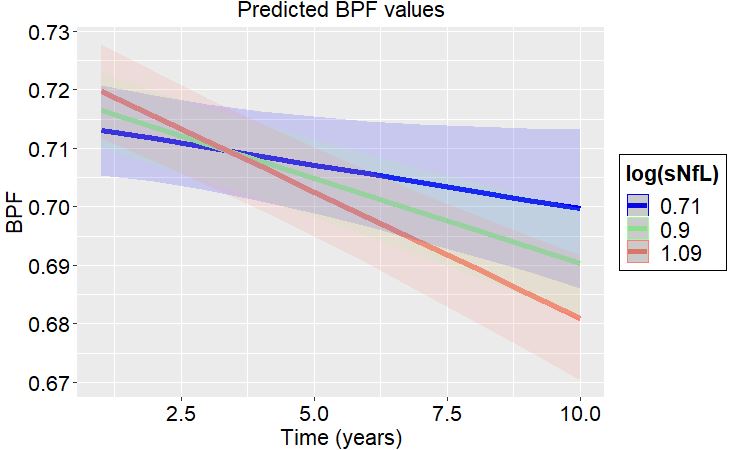
*

**eFigure 3. Interaction effect between serum neurofilament light (sNfL) and time on brain parenchymal fraction (BPF).**

For three Year 1 log-transformed sNfL levels (blue=low, green=middle and orange=high, log-transformed concentrations displayed in the legend), this graph illustrates the direction of the linear-mixed effects on brain parenchymal fraction (BPF). The linear-mixed effects model was corrected for age, disease duration and sex (see Table 2). In conclusion, higher NfL levels are associated with a higher decline in BPF over time (p=0.005).
